# Identification of the novel polymorphisms and potential genetic features of the prion protein gene (*PRNP*) in horses, a prion disease-resistant animal

**DOI:** 10.1038/s41598-020-65731-5

**Published:** 2020-06-02

**Authors:** Yong-Chan Kim, Sae-Young Won, Kyoungtag Do, Byung-Hoon Jeong

**Affiliations:** 1 Korea Zoonosis Research Institute, Jeonbuk National University, Iksan, Jeonbuk 54531 Republic of Korea; 2 Department of Bioactive Material Sciences and Institute for Molecular Biology and Genetics, Jeonbuk National University, Jeonju, Jeonbuk 54896 Republic of Korea; 3 Lab of Equine Science, Department of Animal Biotechnology, Faculty of Biotechnology, Jeju National University, Jeju, 63243 Republic of Korea

**Keywords:** Haplotypes, Population genetics, Genetics, Genetic linkage study, Genotype

## Abstract

Prion diseases, a protein misfolded disorder (PMD) caused by misfolded prion protein (PrP^Sc^), present in a wide variety of hosts, ranging from ungulates to humans. To date, prion infections have not been reported in horses, which are well-known as prion disease-resistant animals. Several studies have attempted to identify distinctive features in the prion protein of horses compared to prion disease-susceptible animals, without the study on polymorphisms of the horse prion protein gene (*PRNP*). Since single nucleotide polymorphisms (SNPs) of *PRNP* in prion disease-susceptible animals are major susceptibility factors, the investigation of SNPs in the horse *PRNP* gene is important; however, only one study investigated a single horse breed, Thoroughbred. Thus, we investigated genetic polymorphisms and potential characteristics of the *PRNP* gene in 2 additional horse breeds. To this end, we performed amplicon sequencing of the horse *PRNP* gene and investigated SNPs in Jeju and Halla horses. We compared genotype, allele and haplotype frequencies among three horse breeds, namely, Thoroughbred, Jeju and Halla horses. In addition, we evaluated the potential influence of the identified nonsynonymous SNPs on the prion protein using PolyPhen-2, PROVEAN, and PANTHER. Furthermore, we measured the aggregation propensity of prion proteins using AMYCO and analyzed linkage disequilibrium (LD) between *PRNP* and *prion-like* protein gene (*PRND)* SNPs. A total of 4 SNPs were found, including two nonsynonymous SNPs (c.301 T > A, c.525 C > A) and three novel SNPs (c.-3A > G, c.301 T > A and c.570 G > A). There were significant differences in genotype, allele and haplotype frequencies among the three horse breeds. The nonsynonymous SNP, c.301 T > A (W101R), was predicted to be benign, deleterious, and possibly damaging by PolyPhen-2, PROVEAN and PANTHER, respectively. In addition, the amyloid propensity of horse prion protein according to 4 haplotypes of nonsynonymous SNPs was predicted to be benign by AMYCO. Finally, we identified weak LD between *PRNP* and *PRND* SNPs.

## Introduction

Prion diseases, which are fatal neurodegenerative diseases and are one type of protein misfolded disorder (PMD) caused by a deleterious form of prion protein (PrP^Sc^) derived from normal prion protein (PrP^C^), are accompanied by spongiform generation and gliosis in brain lesions^1,2^. After scrapie was first discovered in sheep in 1732, it was studied for approximately three centuries. Prion diseases showed a broad infection host ranges, including scrapie in sheep and goats; bovine spongiform encephalopathy (BSE) in cattle; chronic wasting disease (CWD) in elk and deer; transmissible mink encephalopathy (TME) in mink; feline spongiform encephalopathy (FSE) in cats, cheetah and pumas; and Creutzfeldt–Jakob disease (CJD), fatal familial insomnia (FFI) and Gerstmann–Sträussler–Scheinker syndrome (GSS) in humans^3–12^. Although prion diseases have been reported in various host species, a natural case of prion disease in horses, has not been reported to date. Previous studies, including molecular dynamics (MD) and nuclear magnetic resonance (NMR) studies, have attempted to understand the mechanism of prion disease resistance. However, these studies have been conducted in nonpolymorphic prion protein genes (*PRNP*s)^13,14^. Since polymorphisms in the *PRNP* gene significantly affect protein structure and are related to the vulnerability to disease, investigating polymorphisms in the horse *PRNP* gene is very important^15–17^.

In horses, polymorphisms of the *PRNP* gene have been reported in our previous research. Only one single nucleotide polymorphism (SNP), N175K, was found in the open reading frame (ORF) of the horse *PRNP* gene in the Thoroughbred breed^18^. However, since the study was conducted in only Thoroughbred horses, this result is not representative of the genetic diversity of the whole horse species. Thus, we investigated genetic polymorphisms and found genetic characteristics of the *PRNP* gene in Jeju and Halla horses. The Jeju horse is a Korean native horse, and the Halla horse is hybrid between the Jeju and Thoroughbred breeds that is bred for racing purposes.

To achieve these goals, we carried out amplicon sequencing of the horse *PRNP* gene and investigated SNPs in Jeju and Halla horses. In addition, we investigated genotype, allele and haplotype frequencies and compared the distributions among three breeds, Thoroughbred, Jeju and Halla horses. Furthermore, we evaluated the potential influence of the identified nonsynonymous SNPs on the prion protein using PolyPhen-2, PROVEAN, and PANTHER and measured the aggregation propensity of prion protein variants using AMYCO^19–24^. Lastly, we analyzed linkage disequilibrium (LD) between *PRNP* and *prion-like* protein gene (*PRND)* SNPs.

## Results

**Figure 1:**
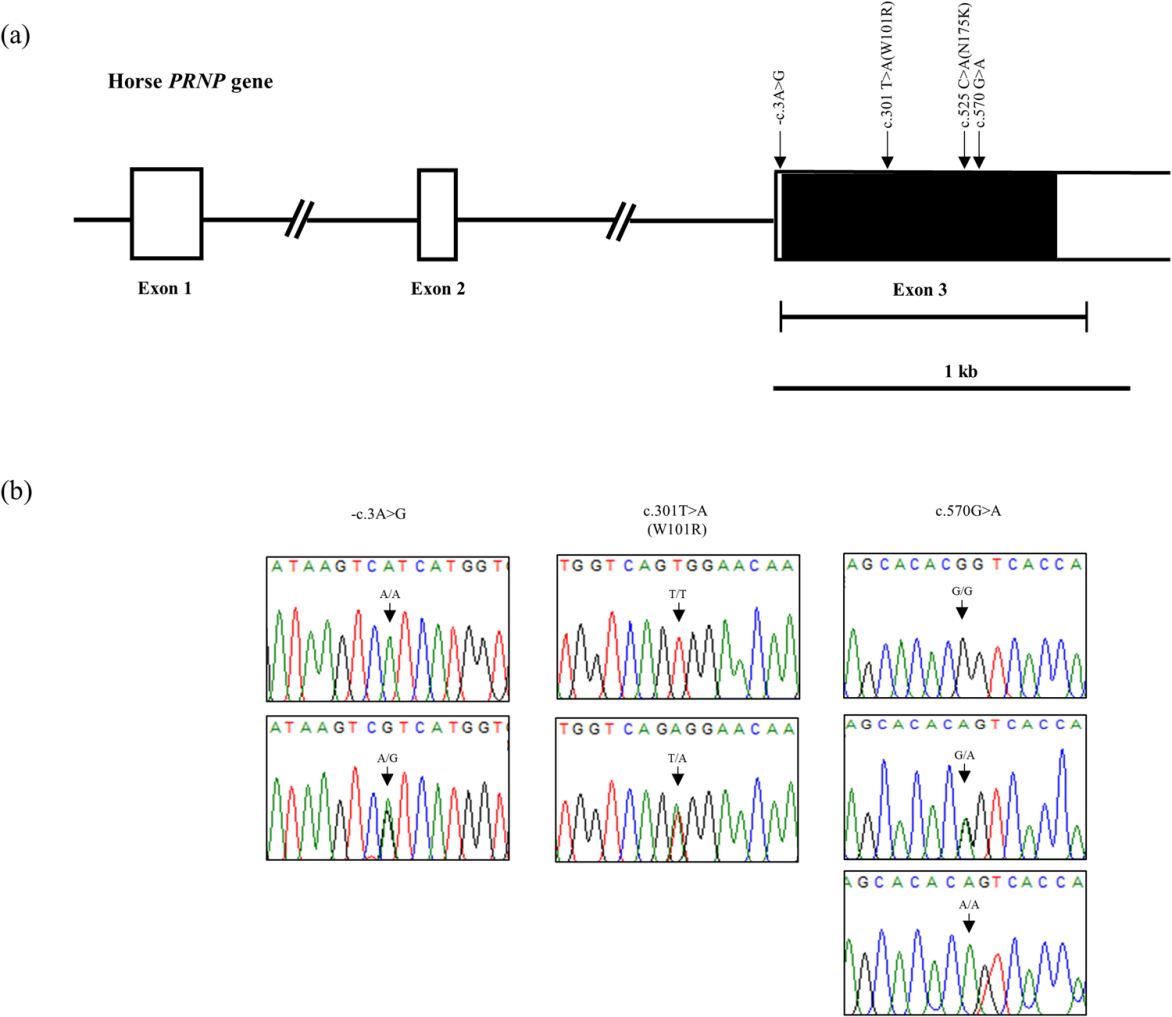
Gene map of and polymorphisms identified in the horse prion protein gene *(PRNP*) on chromosome 22. (**a**) The open reading frame (ORF) within the exons is indicated by shaded blocks, and the 5′ and 3′ untranslated regions (UTRs) are indicated by white blocks. Edged horizontal bars indicate the regions sequenced. Arrows  indicate the polymorphisms found in this study. Asterisks denote novel single nucleotide polymorphisms (SNPs). (**b**) Electropherogram of novel SNPs: c.-3A > G, c.301 T > A (W101R) and c.570 G > A, identified in this study. Four colors indicate individual bases of DNA sequence (blue: cytosine, red: thymine, black: guanine, green: adenine).

The horse *PRNP* gene is composed of three exons. We amplified the ORF region for exon 3 of the *PRNP* gene composed of 836 bp and performed automatic amplicon sequencing in 142 Jeju horses and 82 Halla horses. We found a total of 4 SNPs, including c.-3A > G, c.301 T > A, c.525 C > A and c.570 G > A. Of the 4 SNP, c.301 T > A (W101R) and c.525 C > A (N175K) are nonsynonymous SNPs. Except for c.525 C > A, three SNP, including c.-3A > G, c.301 T > A and c.570 G > A, were novel SNPs found in this study (Fig. 1).

To compare the difference in genetic distribution of SNPs in the horse *PRNP* gene, we performed genotyping and investigated the genotype and allele frequencies of SNPs in this gene (Table 1). Except for c.525 C > A in the Jeju horse, all SNPs are in Hardy-Weinberg Equilibrium (HWE). Then, we compared genotype and allele distributions among the three horse breeds. For c.-3A > G SNP, the Halla horse showed similar genotype and allele distributions as the Jeju horse (P = 1.0). However, Halla horse showed significantly different genotype and allele distributions with Thoroughbred horse (P < 0.0001). For the c.301 T > A SNP, the Jeju horse showed similar genotype and allele distributions with Halla and Thoroughbred horses. Jeju horses showed significantly different genotype and allele distributions with Halla and Thoroughbred horses for c.525 C > A SNP (P < 0.0001). For the 570 G > A SNP, Jeju horses showed significantly different genotype distributions (P = 0.0004) and allele distributions (P = 0.0002) with Halla horses. In addition, Jeju horse also showed significantly different genotype and allele distributions with Thoroughbred horse (P < 0.0001) (Table 1).

**Table 1:** Genotype and allele frequencies of *PRNP* polymorphisms in Jeju, Halla and Thoroughbred horses.

Polymorphism	Breeds	Genotype frequencies			P-value	Allele frequencies		P-value	HWE	Ref
c.-3A > G		AA	AG	GG		A	G			
	Jeju	118 (83.1)	24 (16.9)	0 (0)		260 (91.5)	24 (8.5)		0.271	In this study
	Halla	68 (82.9)	14 (17.1)	0 (0)	1.0	150 (91.5)	14 (8.5)	1.0	0.398	In this study
	Thoroughbred	201 (100)	0 (0)	0 (0)	<0.0001	402 (100)	0 (0)	<0.0001	NA	^18^
c.301 T > A (W101R)		TT	TA	AA		T	A			
	Jeju	140 (98.6)	2 (1.4)	0 (0)	—	282 (99.3)	2 (0.7)	—	0.933	In this study
	Halla	79 (96.3)	3 (3.7)	0 (0)	0.3584	161 (98.2)	3 (1.8)	0.3607	0.866	In this study
	Thoroughbred	201 (100)	0 (0)	0 (0)	0.1707	402 (100)	0 (0)	0.1710	NA	^18^
c.525 C > A ((N175K)		CC	CA	AA		C	A			
	Jeju	79 (55.6)	60 (42.3)	3 (2.1)	—	218 (76.8)	66 (23.2)	—	0.028	In this study
	Halla	28 (34.2)	38 (46.3)	16 (19.5)	<0.0001	94 (57.3)	70 (42.7)	<0.0001	0.632	In this study
	Thoroughbred	11 (5.5)	80 (39.8)	110 (54.7)	<0.0001	102 (25.4)	300 (74.6)	<0.0001	0.470	^18^
c.570 G > A		GG	GA	AA		G	A			
	Jeju	126 (88.7)	16 (11.3)	0 ((0)	—	268 (94.4)	16 (5.6)	—	0.477	In this study
	Halla	57 (69.5)	22 (26.8)	3 (3.7)	0.0004	136 (82.9)	28 (17.1)	0.0002	0.634	In this study
	Thoroughbred	201 (100)	0 (0)	0 (0)	<0.0001	402 (100)	0 (0)	<0.0001	NA	^18^

*NA, Not applicable.

Based on the genotype distributions of horse *PRNP* SNPs, we analyzed the haplotype frequencies of the horse *PRNP* gene in Jeju and Halla horses. A total of 5 haplotypes were identified (Table 2). Significantly different distributions of the haplotypes between these two breeds were found in Haplotype 1 (P = 0.0002), Haplotype 2 (P = 0.0019) and Haplotype 4 (P = 0.0005).

**Table 2:** Haplotype frequencies of 4 *PRNP* polymorphisms in Jeju and Halla horses.

Haplotypes	Polymorphisms				Frequencies		P-value
	c.-3A > G	c.301 T > A	c.525 C > A	c.570 G > A	Jeju	Halla	
Haplotype 1	A	T	C	G	178 (62.7)	52 (31.7)	0.0002
Haplotype 2	A	T	A	G	66 (23.2)	70 (42.7)	0.0019
Haplotype 3	G	T	C	G	22 (7.7)	11 (6.7)	0.7060
Haplotype 4	A	T	C	A	16 (5.6)	28 (17.1)	0.0005
Haplotype 5	G	A	C	G	2 (0.8)	3 (1.8)	0.3629

Next, the extent of LD value was investigated in 4 *PRNP* SNP using Lewontin’s D’ ( | D’ | ) and r^2^ values in Jeju and Halla horses. All four SNPs were strongly linked together with a D’ value of 1.0. However, the LD value among 4 *PRNP* SNPs in the results using the r^2^ value is notably low (below score 0.3) and showed weak LD in Jeju and Halla horses (Tables 3, 4).

**Table 3:** Linkage disequilibrium (LD) among 4 polymorphisms of the *PRNP* gene in Jeju horses.

r^2^	|D’ |			
	c.-3A > G	c.301 T > A	c.525 C > A	c.570 G > A
c.-3A > G	—	1.0	1.0	1.0
c.301 T > A	0.077	—	1.0	1.0
c.525 C > A	0.028	0.002	—	1.0
c.570 G > A	0.006	0	0.018	—

**Table 4:** Linkage disequilibrium (LD) among 4 polymorphisms of the *PRNP* gene in Halla horses.

r^2^	|D’ |			
	c.-3A > G	c.301 T > A	c.525 C > A	c.570 G > A
c.-3A > G	—	1.0	1.0	1.0
c.301 T > A	0.2	—	1.0	1.0
c.525 C > A	0.07	0.025	—	1.0
c.570 G > A	0.019	0.004	0.153	—

We also estimated the potential impact of nonsynonymous SNPs on horse prion protein using PolyPhen-2, PROVEAN and PANTHER. PolyPhen-2 predicted W101R as “Benign” with a value of 0.033. PROVEAN estimated as “Deleterious” with a −2.569 value. PANTHER was assessed as “Possibly damaging” with 361 values (Table 5).

**Table 5:** Prediction of the effect of non-synonymous polymorphism in the horse *PRNP* gene using PolyPhen-2, PROVEAN and PANTHER.

Variation	Method	Score	Prediction
c.301 T > A (W101R)	PolyPhen-2	0.033	Benign
	PROVEAN	−2.569	Deleterious
	PANTHER	361	Possibly damaging

We investigated the amyloid propensity of highly susceptible (ARQ sheep, VRQ sheep, cattle and deer) and non-susceptible (dogs, rabbits and horses) prion proteins using AMYCO. Ovine prion proteins with 136 A/154 R/171Q haplotype and 136 V/154 R/171Q haplotype were measured with 0.27 and 0.31 values, respectively. Bovine and canine prion proteins were measured with 0.00. Deer and rabbit prion proteins were measured with 0.12 and 0.27 values, respectively. Horse prion protein sequences were classified into 4 haplotypes according to alleles of nonsynonymous SNPs (W101R and N175K), and amyloid propensity was analyzed by AMYCO. 101W/175N and 101R/175N haplotypes were measured with 0.4 values. The 101 W/175 K and 101 R/175 K haplotypes were measured with 0.0 values (Table 6).

**Table 6:** Evaluation of the aggregation propensities of prion protein in several species using AMYCO.

Species	AMYCO score	Reference
Sheep, 136A/154R/171Q	0.27	AAB97765.1
Sheep, 136V/154R/171Q	0.31	AAB97765.1
Cattle	0.00	BAA01467.1
Deer	0.12	AAC33174.2
Dog	0.00	AGA63676.1
Rabbit	0.27	AAD01554.1
Horse, 101W/175N	0.39	In this study
Horse, 101W/175K	0.00	In this study
Horse, 101R/175N	0.39	In this study
Horse, 101R/175K	0.00	In this study

**Figure 2:**
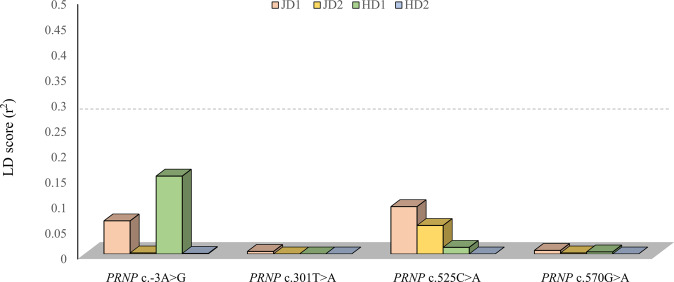
Measurement of LD values between *PRNP* and *PRND* SNPs in Jeju and Halla. horses. JD1 and HD1 indicate horse *PRND* c.331 G > A (codon 111), and JD2 and HD2 indicate horse *PRND* c.411 G > C (codon 137).

To examine whether horse *PRND* SNPs have genetic linkage with the *PRNP* SNPs, we analyzed *PRND* SNPs in 30 Jeju and 30 Halla horses and measured the LD values between SNPs of the *PRNP* and *PRND* genes. Notably, all *PRND* SNP showed weak LD with the *PRNP* SNPs (r^2^ value: blow 0.3; Fig. 2).

## Discussion

In the present study, we validated the polymorphisms in the ORF of the *PRNP* gene in a large sampling of outbred horse breeds, Jeju and Halla horses. Unlike inbred Thoroughbred horses, a total of 4 SNPs, including c.-3A > G, c.301 T > A, c.525 C > A and c.570 G > A, were found in the ORF of the horse *PRNP* gene. Among these SNPs, two (c.301 T > A, c.525 C > A) are nonsynonymous, and three of the four (c.-3A > G, c.301 T > A and c.570 G > A) are novel to this study. Next, we compared genotype and allele distributions among Jeju, Halla and Thoroughbred horses and found significantly different genetic distributions among the horse breeds. Haplotypes of the *PRNP* gene were also analyzed, and a total of 5 haplotypes were identified. In addition, the genetic distributions of haplotypes were significantly different between Halla and Jeju horses. We investigated the genetic linkage among horse *PRNP* SNPs in Jeju and Halla horses. Although we analyzed both parameters (D’ and r^2^ values), D’ value is inflated when one allele is rare. Since 4 *PRNP* SNPs showed that minor alleles are rare, we interpreted the result using r^2^ value. Interestingly, weak LD among horse *PRNP* SNPs was identified in Jeju and Halla horses (Tables 3, 4). In addition, horse showed weak LD between *PRNP* and *PRND* SNPs (Fig. 2). Recent studies have reported that prion disease-susceptible animals, including sheep and goats, showed strong LD between *PRNP* and *PRND* genes^9,25^. On the other hand, in prion disease-resistant animals, dogs showed weak LD between *PRNP* and *PRND* genes, unlike prion disease-susceptible animals^26^.

We also found potential effect of W101R to function of horse prion protein using PROVEAN and PANTHER (Table 5). Next, we estimated amyloid propensity of the horse prion protein. Previous studies have reported that horse prion protein presents a highly stable structure that can endure harsh conditions (high temperature and pH changes). It is suggested that the highly stable structure of the horse prion protein contributes to resistance to conformational changes in the abnormal form of the prion protein, which supports prion disease resistance in horses^27,28^. However, since these studies have not been performed with polymorphisms found in the horse prion protein, these findings are limited. In this study, we performed *in silico* estimation on the amyloid propensity of horse prion proteins with various combinations of the nonsynonymous SNPs. We classified the prion protein sequence into 4 haplotypes based on alleles of nonsynonymous SNPs (W101R and N175K) and performed an analysis of amyloid propensity in horse prion protein. The horse prion protein sequence registered in GenBank (101 W/175 N) had a value of 0.4. Interestingly, the other three haplotypes showed 0.4 (101 R/175 N) and 0.0 (101 W/175 K and 101 R/175 K) values. This result indicates that polymorphisms of horse prion protein showed no effect on amyloid propensity in the 101 R/175 N haplotype were unlikely to form amyloid while the 101 W/175 K and 101 R/175 K haplotypes. Although amyloid propensity prediction score did not strictly correlate with the susceptibility of prion disease (cattle: 0.00; rabbits: 0.27), the amyloid propensity of equine prion protein was predicted to be different according to alleles of nonsynonymous SNPs (Table 6). Further studies using *in vivo* and *in vitro* models are warranted.

In conclusion, we investigated prion genetic characteristics in horses and validated the polymorphisms in the ORF of the *PRNP* gene in a large sampling of outbred horse breeds, Jeju and Halla horses. In addition, we found significantly different genetic distributions of genotype, allele, and haplotype in three horse breeds in the *PRNP* gene and identified significantly weak LD between *PRNP* and *PRND* SNPs, unlike those of prion disease-susceptible animals. Furthermore, we evaluated the amyloid propensity of horse prion protein according to alleles of nonsynonymous SNPs. To the best of our knowledge, we analyzed potential impact of novel polymorphisms of the horse *PRNP* gene with the regard to susceptibility to prion disease. The weak LD results between horse *PRNP* and *PRND* SNPs correspond well with results from other prion disease resistant species. High amyloid propensity values do not align with prion disease susceptible species making the results generated for horse PrP difficult to interpret making further examination results.

## Methods

### Ethics statement

We extracted DNA from hair samples of 148 Jeju horses and 100 Halla horse horses in Jeju Island. All experimental procedures were approved by the Institute of Animal Care and Use Committee of Chonbuk National University (CBNU 2016-65). All experiments using horses were carried out following the Korea Experimental Animal Protection Act.

### Genetic analysis

Genomic DNA was obtained from 10 hair bulbs using the HiYieldTM genomic DNA mini kit (Real Biotech Corporation, Taiwan) according to manufacturer’s recommendations. The horse *PRNP* and *PRND* were amplified from the genomic DNA using sense and antisense gene-specific primers. The DNA sequences of the primers were as follows: Horse PRNP-F (AGAAGTGCAGAGTGTGACATGC), Horse PRNP-R (CAAGCGTATTAGCCTACGGGTG), Horse PRND-F (GCCCGTTGCAGCTTCTTATCT) and Horse PRND-R (GCTGGAGGAGAGAAGTGGGAT). Polymerase chain reaction (PCR) was carried out using GoTaq® DNA Polymerase (Promega, Fitchburg, Wisconsin, USA) as described previously^9^. In brief, the PCR mixture consisted of 20 pmol of each primer, 5 μl of 10× *Taq* DNA polymerase buffer, 1 μl of 10 mM dNTP mixture and 2.5 units of *Taq* DNA polymerase. The PCR conditions were 94 °C for 2 min for denaturation; 35 cycles of 94 °C for 45 sec, 59 °C for 45 sec, and 72 °C for 1 min 30 sec; and then 1 cycle of 72 °C for 10 min to extend the reaction. PCR was performed using an S-1000 Thermal Cycler (Bio-Rad, Hercules, California, USA). The PCR products were purified by the PCR Purification Kit (Thermo Fisher Scientific, Bridgewater, New Jersey, USA) and 5 μl of purified PCR product (50 ng/μl) was sequenced with an ABI 3730 automatic sequencer (ABI, Foster City, California, USA) using 5 pmol of sense and antisense primers as described previously^10^. Sequencing results were analyzed by Finch TV software (Geospiza Inc., Seattle, USA).

### Statistical analysis

Comparisons of genotype, allele and haplotype frequencies were performed by the chi-square test using SAS 9.4 Software (SAS Institute Inc., Cary, NC, USA) as described previously^26^. The HWE and haplotype analyses were performed using Haploview version 4.2 (Broad Institute, Cambridge, MA, USA). LD analysis was carried out between *PRNP* and *PRND* SNPs. LD score of the *PRNP* and *PRND* genes were calculated in 30 Jeju horses and 30 Halla horses using Haploview version 4.2 (Broad Institute, Cambridge, MA, USA) as described previously^3^.

### Evaluation on potential impact of nonsynonymous SNP to horse prion protein

The biological impact of horse prion protein induced by the nonsynonymous SNPs was assessed by PolyPhen-2 (http://genetics.bwh.harvard.edu/pph2/index.shtml), PROVEAN (http://provean.jcvi.org/seq_submit.php) and PANTHER (http://www.pantherdb.org/) as described previously^24^. PolyPhen-2 uses a position-specific, independent count (PSIC) score (score ranges from 0.0 to 1.0). The prediction results can be presented as three types: ‘benign’, ‘possibly damaging’ and ‘probably damaging’^21^. PROVEAN is a program that measures the impact of nonsynonymous SNPs on protein function. If the final score is below −2.5, protein variants are predicted to be ‘neutral’, and if the final score is above −2.5, protein variants are predicted to be ‘deleterious’^22^. PANTHER utilizes a hidden Markov model (HMM) based on statistical modeling methods and multiple sequence alignments to perform evolutionary analysis of nonsynonymous SNPs. PANTHER predicts SNPs as two types: ‘deleterious’ or ‘neutral’^23^. Amyloid propensity of horse prion protein according to alleles was analyzed by AMYCO (http://bioinf.uab.cat/amyco) as described previously^24^. AMYCO utilizes an algorithm to predict amyloid fibril propensity from amino acid sequences^19^.
